# 17-β Estradiol Rescued Immature Rat Brain against Glutamate-Induced Oxidative Stress and Neurodegeneration via Regulating Nrf2/HO-1 and MAP-Kinase Signaling Pathway

**DOI:** 10.3390/antiox10060892

**Published:** 2021-06-01

**Authors:** Ibrahim Khan, Kamran Saeed, Min Gi Jo, Myeong Ok Kim

**Affiliations:** Division of Life Sciences and Applied Life Science (BK 21 FOUR), College of Natural Science, Gyeongsang National University, Jinju 52828, Korea; ibrahim1994@gnu.ac.kr (I.K.); kamran.biochem@gnu.ac.kr (K.S.); mingi.cho@gnu.ac.kr (M.G.J.)

**Keywords:** 17β-estradiol, glutamate, oxidative stress, MAP-kinases, neuroinflammation, gliosis, neurodegeneration

## Abstract

Dysregulated glutamate signaling, leading to neuronal excitotoxicity and death, has been associated with neurodegenerative pathologies. 17β-estradiol (E2) is a human steroid hormone having a role in reproduction, sexual maturation, brain health and biological activities. The study aimed to explain the neuroprotective role of E2 against glutamate-induced ROS production, MAP kinase-dependent neuroinflammation, synaptic dysfunction and neurodegeneration in the cortex and hippocampus of postnatal day 7 rat brain. Biochemical and immunofluorescence analyses were applied. Our results showed that a single subcutaneous injection of glutamate (10 mg/kg) induced brain oxidative stress after 4 h by disturbing the homeostasis of glutathione (GSH) and revealed an upsurge in ROS and LPO levels and downregulated the expression of Nrf2 and HO-1 antioxidant protein. The glutamate-exposed P7 pups illustrated increased phosphorylation of stress-activated c-Jun N-terminal kinase (JNK) and p38 kinase (p38) and downregulated expression of P-Erk1/2. This was accompanied by pathological neuroinflammation as revealed by enhanced gliosis with upregulated expression of GFAP and Iba-1, and the activation of proinflammatory cytokines (TNF-α) in glutamate-injected P7 pups. Moreover, exogenous glutamate also reduced the expression of synaptic markers (PSD-95, SYP) and induced apoptotic neurodegeneration in the cortical and hippocampal regions by dysregulating the expression of Bax, Bcl-2 and caspase-3 in the developing rat brain. On the contrary, co-treatment of E2 (10 mg/kg) with glutamate significantly abrogated brain neuroinflammation, neurodegeneration and synapse loss by alleviating brain oxidative stress by upregulating the Nrf2/HO-1 antioxidant pathway and by deactivating pro-apoptotic P-JNK/P-p38 and activation of pro-survival P-Erk1/2 MAP kinase pathways. In brief, the data demonstrate the neuroprotective role of E2 against glutamate excitotoxicity-induced neurodegeneration. The study also encourages future studies investigating if E2 may be a potent neuroprotective and neurotherapeutic agent in different neurodegenerative diseases.

## 1. Introduction

Glutamate is a free amino acid, rich in CNS and known as a major excitatory neurotransmitter, connected to essentially most activities of the nervous system [1], including fast synaptic communication, neuronal plasticity, survival and outgrowth [2]. However, under pathological conditions, glutamate plays a crucial role in neuronal cell death [3], and its dysregulation has been reported in both acute and chronic neurodegenerative disease, including ischemia, amyotrophic lateral sclerosis, Huntington’s disease and Alzheimer’s disease (AD) [1,4]. The over-activation of NMDA receptors due to excessive glutamate can mediate abnormal Ca^2+^ influx, increase cellular reactive species generation (ROS) and damages mitochondrial membrane, inducing neuronal apoptosis and cell death [5,6]. Therefore, the neuroprotection against glutamate-induced excitotoxicity may present a promising therapeutic strategy in alleviating acute/chronic neurodegenerative disease, at least to delay their onset/appearance [7].

Prolonged glutamate excitotoxicity is known to induce cystine depletion, leading to deficiency in the cellular store of glutathione, causing increase oxidative stress (OS) [8]. OS plays an important role in regulating the cellular redox state, contributing to ROS generation, which are the main culprits in promoting neurodegenerative disease [9]. ROS has deleterious effects on post-mitotic neuronal and glial cells, leading to programmed cell death/apoptosis [10]. In neuronal tissue and brain, ROS in particularly are active due to the unique brain metabolism of neurotransmitters and excitatory amino acids and serve as a source of OS [11]. The normal brain has an antioxidant defense mechanism against increased OS to regulate cellular redox homeostasis. Nrf2, an important transcription factor, recognizes antioxidant response element (ARE) to encode key cytoprotective enzymes such as glutathione peroxidase 1 (GPx1), heme oxygenase 1 (HO-1) and SOD1 [12] and also regulates endogenous antioxidant genes, including glutathione (GSH), to promote cell survival against brain OS [13].

ROS-induced oxidative damage is a risk factor in the development of many neurodegenerative diseases [14] and is involved in the potential activation of the MAPKs (serine-threonine protein kinases) pathway. MAPKs cascades regulate numerous cellular activities, including cell survival/death and proliferation/differentiation [15]. The oxidative modification due to increased ROS generation leads to regulation of MAPK/extracellular signal-regulated kinase (Erk), c-jun NH2-terminal kinases (JNKs) or p38 MAPKs. Where the Erk pathway is associated with cell survival, the stress-activated JNK and p38-MAPK pathways are associated with cell death [16]. MAPKs activation is responsible for astrogliosis and microgliosis and induces the production of proinflammatory mediators such as tumor necrosis factor-α (TNF-α), interleukin-1β (IL-1β) and cyclooxygenase-2 (COX-2) [17,18]. Excessive glutamate-induced OS may activate the p38 MAPK pathway and culminate with glial and neuronal cell death and apoptosis [19].

17β-estradiol or estradiol (E2) exists as human steroid hormones having a role in reproduction, sexual maturation, lipid metabolism, cardiovascular system and brain health [20,21,22,23]. In addition to the importance of sexual characteristics, E2 is also crucial for fetal and embryonal development of the brain networks [24]. E2 is considered as a neurosteroid hormone, which is synthesized in the brain and is released to participate in numerous signaling pathways [25,26,27]. Many studies have illustrated the neuroprotective role of E2 [28,29,30,31]. Accumulative studies have shown the beneficial outcome of E2 in different CNS injuries, including ischemic brain injury (IBI), spinal cord injury (SCI) and traumatic brain injury (TBI) [32]. E2 mediates signaling pathways mostly through estrogen receptors (ERs), either through genomic or non-genomic mechanism [33]. However, many studies have demonstrated the ER-independent antioxidant effect of estrogens [34,35] to protect different neuronal cell lines against oxidative stress induced by superoxide anions, hydrogen peroxide and other pro-oxidants [36]. E2-mediated protection against oxidative cell death induced by glutamate or amyloid-β was largely mitigated by pre-treatment neuronal cells with MEK inhibitor PD98059, thus showing clearly the activation of Erk by E2. Moreover, E2 can potentially activate Erk phosphorylation in the HT-22 mouse hippocampus cell line, overexpressing ERα and ERβ [37,38]. Previously, we showed that E2 modulates Sirt1 activities to impede oxidative stress-mediated cognitive decline in the ageing mouse model [39]. Growing evidence also suggests the role of E2 and ERs ligands in exerting anti-inflammatory effects in different models [40,41,42,43,44]. E2 replacement therapy has been used clinically to improve cognitive performance and to reduce the risk of developing AD in women after menopause [45,46]. Here, we examined for the first time the neuroprotective role of E2 in vivo against glutamate-induced oxidative stress, neuroinflammation and neurodegeneration. Our results make evident that glutamate (subcutaneous injection; 10 mg/kg) excitotoxicity compromises the intracellular redox state, disrupting Nrf2/HO-1 protein expressions enhancing ROS and mediating the P-JNK/P38 MAPK signaling pathway, leading to neuroinflammation, neuronal apoptosis and synapse loss in postnatal day 7 (P7) rat brains. However, co-treatment of E2 (subcutaneous injection; 10 mg/kg) with glutamate alleviated oxidative stress, neuroinflammation and neurodegeneration and improved the expression levels of synaptic protein.

## 2. Materials and Methods

### 2.1. Chemicals

Glutamate (Glu) and 17β-estradiol (E2) were purchased from Sigma Aldrich (St. Louis, MO, USA and Madison, WI, USA, respectively).

### 2.2. Animals and Drug Treatment

The postnatal day 7 Dawley male rat pups (16–18 g body weight) were randomly divided into four groups (*n* = 6 pups/group):

(1) Control group (treated subcutaneously with 0.9% saline as a vehicle)
(2) Glu group (treated subcutaneously with 10 mg/kg of glutamate)
(3) Glu + E2 group (co-treated subcutaneously with 10 mg/kg of glutamate plus 10 mg/kg of 17β-estradiol).
(4) E2 group (treated subcutaneously with 10 mg/kg of 17β-estradiol)

**Figure 1:**
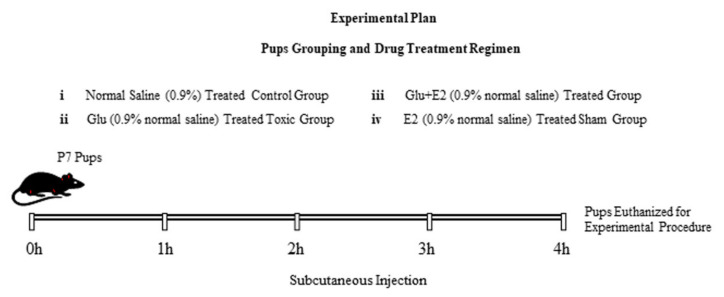
Schematic representation of the research plan. The experimental postnatal day 7 (P7) rat pups were randomly divided into four groups: (i) Pups treated with normal saline as a vehicle (on day 7 subcutaneous injection); normal saline-treated (Cont.) group. (ii) Pups treated with glutamate (subcutaneous injection: 10 mg/kg); glutamate-alone-treated group. (iii) Pups treated with glutamate (10 mg/kg) + E2 (10 mg/kg) subcutaneous injection: Glut + E2 co-treated group. (iv) Pups treated with E2 (subcutaneous injection: 10 mg/kg): E2-alone-treated group. Four hours after a single injection, the experimental pups were euthanized and further subjected to biochemical and immunofluorescence analyses.

Four hours after single subcutaneous injections, all the rat pups were euthanized and sacrificed for biochemical and immunohistochemical analysis. The schematic (Figure 1) illustrates the study design and treatment plan. The experimental procedures were approved (Approval ID: 125) by the local animal ethics committee (IACUC) of the Division of Applied Life Sciences, Department of Biology, Gyeongsang National University, South Korea.

### 2.3. Brain Tissue Collection/Sample Preparation

On postnatal day 7, Dawley (male) rat pups were sacrificed after 4 h of injection, and the brain was removed immediately for biochemical analysis and stored at −80 °C. For immunoblot analysis, tissues were homogenized in 0.2 M phosphate-buffered saline (PBS) with phosphatase inhibitor and protease inhibitor cocktail. The sample was centrifuged at 13,000 rpm at 4 °C for 20 min, and the supernatant was collected and stored at −80 °C. For immunofluorescence analysis, the animals anaesthetized were perfused transcardially with normal saline solution until the whole blood from the body was removed followed by fixation with 4% paraformaldehyde (PFA). The brain was then removed, fixed in PFA at 4 °C for 72 h and then kept in 20% sucrose solution for 48 h and frozen in O.C.T. (TissueTek O.C.T. Compound Medium, Sakura Finetek USA, Inc., Torrance, CA, USA). For the preparation of brain slices, the coronal plane (14 μm) tissue sections were obtained and thaw-mounted on the gelatin-coated slide using a CM 3050C cryostat (Leica, Germany).

### 2.4. Western Blot Analysis

The Bio-Rad protein assay (Bio-Rad Laboratories, Hercules, CA, USA) was used to quantify protein concentration. An equal amount of protein sample (20 mg) was electrophoresed on 12% SDS-PAGE gel and then transferred to polyvinylidene fluoride (PVDF) membrane. For covering a broad range of molecular weights, the pre-stained protein ladder (GangNamstainTM, iNtRON Biotechnology, Burlington, NJ, USA) was used to detect the molecular weights of the proteins. Five percent skim milk was used for membrane blocking to reduce nonspecific binding, followed by incubation with primary antibodies of interest at 4 °C overnight. After incubation, the membranes were washed with 1× TBST and blocked with horseradish peroxidase-conjugated secondary antibody as appropriate. After washing, the bands were detected using an enhanced chemiluminescent (ECL) detection reagent (EzWestLumiOne, ATTO, Tokyo, Japan). The optical densities of the bands were evaluated using ImageJ (v. 1.50, NIH, Bethesda, MD, USA) software.

### 2.5. Immunofluorescence

Immunofluorescence staining was performed as described previously [47]. The slides were washed with 1× PBS followed by incubation at room temperature with proteinase K solution and blocked with normal goat serum. The slides were then incubated with primary antibodies (1:100 in PBS) at 4 °C overnight. The next day, the secondary antibodies fluorescence-based (IgG-FITC/TRITC from Santa Cruz Biotechnology) were applied for 1 h at room temperature. Finally, 4,6-diamidino-2-phenylindole (DAPI) was used for nucleus staining, and the slides were then covered by coverslips with the help of a mounting medium. For immunofluorescence microscopic images, a confocal microscope (FluoView FV 1000; Olympus, Tokyo, Japan) was used. P-JNK, caspase-3 and GFAP cell bodies (stained with DAPI) were manually counted and reviewed morphologically.

### 2.6. Antibodies

The following primary antibodies were used in this study (Table 1), while goat anti-mouse and goat anti-rabbit horseradish peroxidase (HRP) were used as secondary antibodies (dilution 1:10,000), purchased from Santa Cruz Biotech (Dallas, TX, USA) and Cell Signaling Technology (Danvers, MA, USA).

### 2.7. GSH Assays

A glutathione assay kit (BioVision Incorporated155 S. Milpitas Boulevard, Milpitas, CA 95035, USA) and fluorometric assay kit (catalogue #K264-100) were used to measure the total level of GSH and GSH/GSSG enzyme levels according to the manufacturer’s instructions.

### 2.8. Reactive Oxygen Species (ROS) Assay

For ROS detection, the brain homogenates were diluted in ice-cold Lock’s buffer at a 1:20 to make the final concentration of 2.5 mg tissue/500 µL. The final 1 mL reaction mixture composed of Lock’s buffer (pH ± 7.4), brain homogenate 0.2 mL and 10 mL of DCFH-DA (5 mM) was incubated for 15 min to convert DCFH-DA into the fluorescent product DCF at room temperature. A spectrophotometer (Promega Biosciences, CA, USA, excitation at 484 nm and emission at 530 nm) was used to measure the fluorescent product DCF.

### 2.9. Lipid Peroxidation (LPO) Assay

The LPO levels were investigated by quantification of malondialdehyde (MDA) contents. The colorimetric/fluorometric assay kit was used to measure MDA levels according to the manufacturer’s instructions (Bio Vision, San Francesco, CA, USA, Cat #739-100).

### 2.10. Statistical Analyses

For the immunoblot, the band’s densities of the scanned X-ray films were measured and analyzed via ImageJ software (v. 1.50, NIH, Bethesda, MD, USA). Immunofluorescence analysis was either analyzed by integrated densities using ImageJ or by manually counting the number of positive cells stained with DAPI. The data were presented as the mean ± standard error of the mean (SEM). One-way analysis of variance (ANOVA) with Tukey’s post hoc test was used for statistical analysis/significance (*p*-value) using GraphPad Prism 6 (GraphPad Software, San Diego, CA, USA). *p* < 0.05 was considered significant. * *p* < 0.05, ** *p* < 0.01 indicates the comparison between control and Glu-treated groups; # *p* < 0.05, ## *p* < 0.01 indicates the comparison between Glu-treated and E2 + Glu-treated groups, where *p* > 0.05 represents a non-significant (n.s) value. 

## 3. Results

### 3.1. 17β-Estradiol Reduced Glutamate-Induced Oxidative Stress by Activating Nrf2/HO-1 Pathway and Enhanced Cellular Glutathione Stores in Postnatal Rat Brain

**Figure 2:**
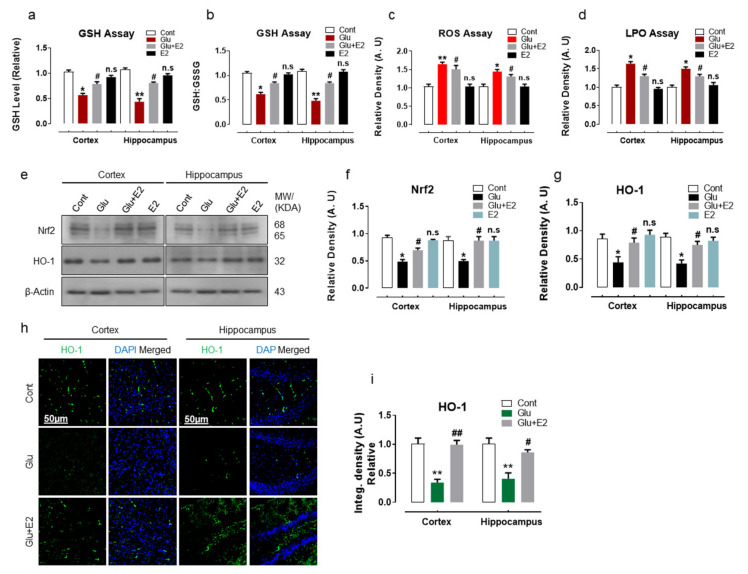
17β-Estradiol turndown ROS production in glutamate-treated postnatal rat brain. Representative histograms show (**a**) GSH level, (**b**) GSH:GSSG enzymes ratio, (**c**) ROS level and (**d**) LPO assay levels in the P7 pups (*n* = 6 rats/group) of brain homogenates. (**e**–**g**) Western blot analysis with their respective histograms of Nrf2 and HO-1 in the brain homogenates of the developing rat brain. The bands were quantified by using ImageJ software; the differences were represented by histograms. β-actin was used as a loading control. Statistical analysis was done through one-way ANOVA. The density values are expressed in arbitrary units (A.U.) as the means ± S.E.M. for the respective indicated protein. (**h**,**i**) Confocal microscopy of HO-1 (green) with respective histogram stained with DAPI (blue) in cortex and hippocampus (DG region) of the postnatal brain. Data are presented relative to the control. Magnification 10×. Scale bar = 50 μm. Significance * *p* < 0.05, ** *p* < 0.01 vs control group and ^#^ *p* < 0.05, ^##^ *p* < 0.01 vs glutamate-injected group. n.s = non-significant difference.

Glutamate plays an important role in neuronal excitotoxicity and mediates reactive oxygen species (ROS) production [5,48]. Overwhelming production of ROS disrupts the balance between pro-oxidants and antioxidants, causing alterations in cellular redox homeostasis, leading to oxidative stress, which in turn has been implicated in many neurodegenerative diseases [49,50]. In this study, we have demonstrated that the subcutaneous injection of glutamate (10 mg/kg) to postnatal day 7 (P7) rat significantly depleted the cellular-store of reduced glutathione (GSH) and GSH to oxidized glutathione (GSH:GSSG) ratio in the cortex and hippocampal brain homogenates as revealed by GSH assay when compared to control littermates (Figure 2a,b). Moreover, the reduced GSH levels in glutamate-injected P7 pups were associated with enhanced ROS and lipid peroxidation (LPO) production as revealed by increased levels of DCF fluorescence and MDA contents, respectively, compared to the non-treated wild group (Figure 2c,d). Furthermore, the immunoblot results also revealed alterations in the endogenous antioxidant pathway in glutamate-injected pups, as indicated by the suppression of Nrf2 and HO-1 protein expressions in the cortical and hippocampal brain homogenates when compared to the control pups (Figure 2e–g). The immunofluorescence analysis of the brain slices further invigorated the disruption of HO-1 enzyme expression in the cortex and within the hippocampal-DG region of the glutamate-injected postnatal rat compared to the non-treated P7 group (Figure 2h,i). However, 17β-estradiol (E2), when co-injected subcutaneously (10 mg/kg) with glutamate, significantly upregulated the Nrf2 protein expression and HO-1 expression and immunoreactivity (Figure 2e–i) and also prominently enhanced the GSH and GSH:GSSG enzyme ratio and reduced the ROS and MDA content in cortical and hippocampal brain regions (Figure 2a–d). Notably, the treatment of E2-alone had no significant effect on the studied parameters when compared to the saline-treated control group (Figure 2a–g).

These results demonstrate that glutamate administration disrupts cellular redox homeostasis, leading to increased brain oxidative stress, while the estradiol treatment could protect against the glutamate-induced neurotoxicity by improving the endogenous antioxidant activity through regulating cytoprotective enzymes (GSH and HO-1) in postnatal rat brain.

### 3.2. 17β-Estradiol Alleviates Glutamate Induced Neuroinflammation in Developing Rat Brain

**Figure 3:**
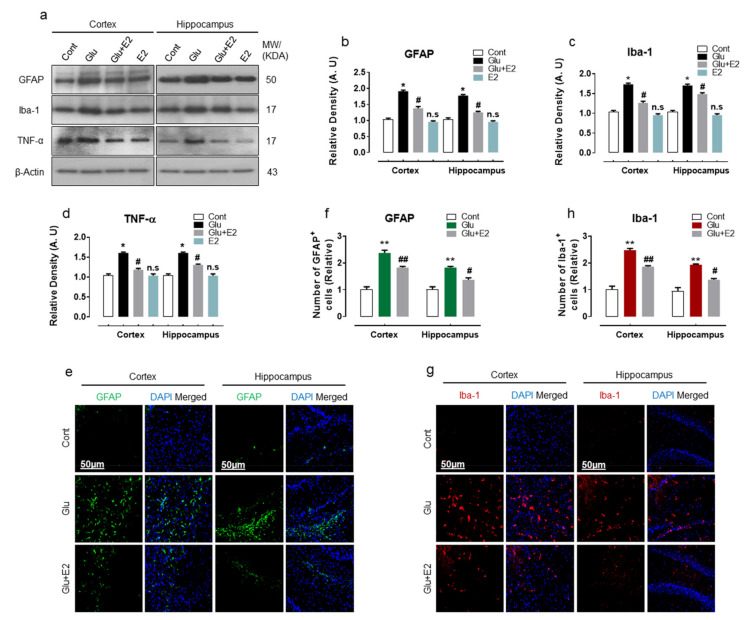
17β-Estradiol treatment attenuates neuroinflammation in postnatal day 7 rat brain. (**a**–**d**) Western blot bands with their respective histograms of GFAP, Iba-1 and TNFα in the cortex and hippocampus of postnatal day 7 rats (*n* = 6 rats/group) brain. Bands were quantified by ImageJ software; the differences were represented by a histogram. β-actin was used as a loading control. Statistical analyses were performed by one-way ANOVA. Arbitrary units (A.U.) were used to express density values and means ± S.E.M. for the respective indicated protein. (**e**–**h**) Immunofluorescence analysis of GFAP (green) and Iba-1 (red) stained with DAPI (blue) with a respective histogram showing no. of positive cells in the cortex and hippocampus (DG region) in the P7 brain. The data are presented relative to control. Magnification 10×. Scale bar = 50 µm. Significance * *p* < 0.05, ** *p* < 0.01 vs control group and ^#^ *p* < 0.05, ^##^ *p* < 0.01 vs glutamate-injected group. n.s = non-significant difference.

Accumulative evidence revealed that glutamate-induced excitotoxicity enhances glial cell activation and promotes neuroinflammatory response in the brain [51,52]. Being an imperative mediator of neuroinflammation, we analyzed the expression of GFAP, Iba-1 and tumor-necrosis factor (TNF-α) protein expressions in the cortex and hippocampal brain regions of the experimental animals. Compared to WT normal pups, the glutamate-injected P7 rat brain displayed a significant induction of gliosis as revealed by increased expression of GFAP and Iba-1 proteins and was also accompanied by the upregulated expression of pro-inflammatory cytokine TNF-α (Figure 3a–d). Immunofluorescence analysis further confirmed the induction of gliosis in the brain slices of glutamate-injected rat brain, as illustrated by the increased number of positive GFAP and Iba-1 cell in the cortex and hippocampal-DG region (Figure 3e–h). In contrast, co-treatment of E2 with glutamate alleviated brain inflammation as revealed by decreased expression of GFAP, Iba-1 and TNF-α (Figure 3a–d). The brain slices of E2 + glutamate-treated pups also displayed a reduced number of GFAP and Iba-1-positive cell bodies within the cortex and hippocampal-DG region (Figure 3e–g). These data illustrate that glutamate-induced oxidative damage is accompanied by neuroinflammation, and treatment with E2 could protect the developing brain against the neuroinflammatory mediator associated with the pathological state.

### 3.3. 17β-Estradiol Abrogates Glutamate-Induced Synaptic Dysfunction in Postnatal Rat Brain

**Figure 4:**
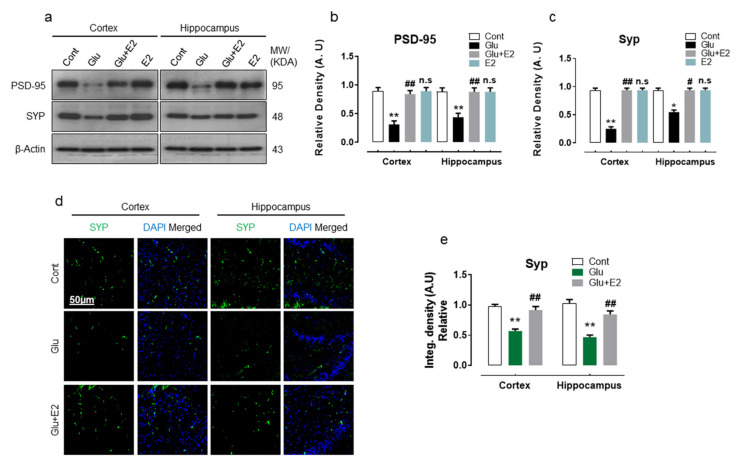
17β-Estradiol inhibited synaptic dysfunction in postnatal day 7 rat brain. (**a**–**c**) Immunoblot with their respective histograms of PSD-95 and SYP in the cortex and hippocampus of postnatal rats group (*n* = 6 rats/group). Bands were quantified by ImageJ software; the differences were represented by a histogram. β-actin was used as a loading control. Statistical analysis was performed via one-way ANOVA. The density values are expressed in arbitrary units (A.U.) and mean ± S.E.M. for the respective indicated protein. (**d**,**e**) Immunofluorescence analysis of SYP (green) with respective histogram stained with DAPI (blue) within the cortex and hippocampal-DG region. The data are presented relative to control. Magnification 10×. Scale bar = 50 µm. Significance * *p* < 0.05, ** *p* < 0.01 vs control group and ^#^ *p* < 0.05, ^##^ *p* < 0.01 vs glutamate-injected group. n.s = non-significant difference.

Glutamate-induced neuronal toxicity causes synaptic dysfunction [53,54], which is a primordial step in mediating the cascade of neuropathological events associated with many neurodegenerative disorders [55]. Accordingly, we observed that glutamate-injected pups compared to their normal counterparts had reduced expression of presynaptic protein synaptophysin (SYP) and postsynaptic density protein 95 (PSD95) in cortical and hippocampal homogenates gauged by Western blot analysis (Figure 4a–c). Moreover, the confocal imaging of the brain slices of glutamate-injected rat pups also revealed the loss of SYP immunoreactivity in the cortex and within the hippocampal-DG region (Figure 4d,e). Conversely, E2 co-treatment significantly attenuated the synapse loss by perpetuating SYP and PSD-95 protein expression in glutamate-injected rat brain (Figure 4a–e).

These data illustrate that exogenous glutamate administration instigates synaptotoxicity by downregulating the expressions of pre-and post-synaptic protein, while E2 treatment may potentially restrain the glutamate-induced synapse loss.

### 3.4. 17β-Estradiol Treatment Overcame Glutamate-Induced Neurodegeneration in Postnatal Rat Brain

**Figure 5:**
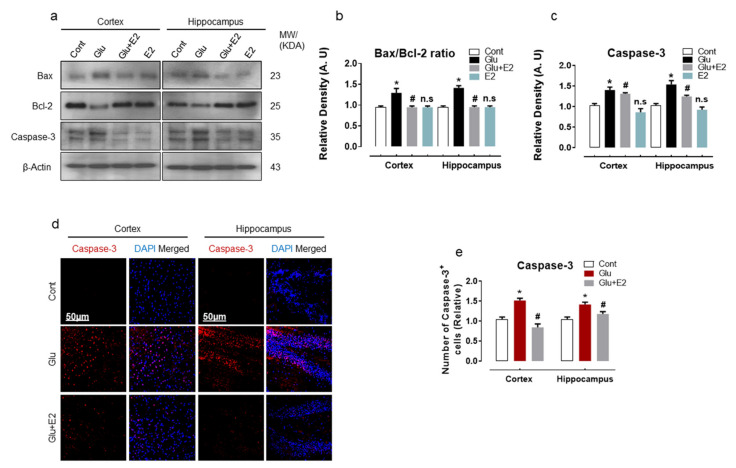
17β-Estradiol alleviates neurodegeneration in postnatal rat brain. (**a**–**c**) Immunoblot analysis with their respective histograms of Bax/Bcl-2 and Caspas3 proteins in the cortex and hippocampus of the P7 rat brain homogenates (*n* = 6 rats/group). Bands were quantified by ImageJ software. The differences were represented by a histogram. β-actin was used as a loading control. One-way ANOVA was used to determine statistical significance between the groups. The arbitrary units (A.U.) were used to express the density values and means ± S.E.M. for the respective indicated protein. (**d**,**e**) Immunofluorescence analysis of Caspas3 (red) stained with DAPI (blue) with a respective histogram showing no. of positive caspase-3 cells in the cortex and hippocampus (DG region) of P7 brain slices. The data are presented relative to control. Magnification 10×. Scale bar = 50 µm. Significance * *p* < 0.05 vs control group and ^#^ *p* < 0.05 vs glutamate-injected group. n.s = non-significant difference.

Glutamate-induced oxidative damages are associated with the disruption of mitochondrial membrane potential and initiate the cascade of the pro-death process contributing to cell death in many neurodegenerative diseases [56,57]. To analyze glutamate-induced cell death and neurodegeneration, we examined Bax, Bcl2 and caspase-3 protein expression in the P7 brain. Immunoblot quantification revealed an increased Bax/Bcl-2 ratio and caspase-3 expression in the cortex and hippocampal brain homogenates of glutamate-injected rats when compared to the control group (Figure 5a–c). Confocal microscopy further corroborated the evidence of caspase-3 activation in brain slices of the glutamate-injected pups (Figure 5d,e). However, co-administering E2 with glutamate curtailed the expression of Bax/Bcl2 protein (Figure 5a,b). Moreover, the treatment with E2 also suppressed caspase-3 expression (Figure 5a,b) and reduced the number of positive caspase-3 cell bodies (Figure 5d,e), as indicated by immunoblot and immunofluorescence analysis, respectively, in the cortex and hippocampal brain regions.

Overall, these results suggest that exogenous glutamate has a deleterious effect on mitochondrial functions in the developing brain and mediates the execution of apoptotic cell death and neurodegeneration, whilst the E2 can protect the brain against glutamate-induced neuronal apoptosis and cell death.

### 3.5. 17β-Estradiol Protect Developing Rat Brain against Glutamate-Induced Excitotoxicity through Regulating P-JNK/P38 and Erk1/2 MAPK Signaling Pathways

**Figure 6:**
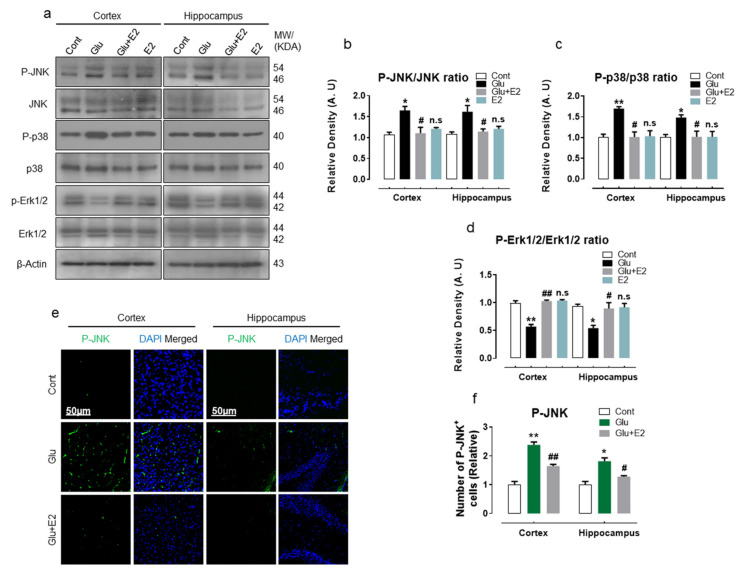
17β-Estradiol regulates the activation of glutamate-induced MAP kinases. (**a**–**d**) Immunoblot analysis with their respective histograms of P-JNK/JNK, P-p38/p38 and P-Erk/Erk proteins of the experimental groups (*n* = 6 rats/group) of the postnatal rat brain. ImageJ software was used for band quantification; the differences were represented by a histogram. β-actin was used as a loading control. One-way ANOVA was used for statistical analysis. Arbitrary units (A.U.) were used to expressed density values and means ± S.E.M. for the respective indicated protein. (**e**,**f**) Immunofluorescence analysis of p-JNK (green) stained with DAPI (blue) with a respective histogram showing no. of positive P-JNK cells in the cortex and hippocampus of the DG region. The data were presented relative to control. Magnification 10×. Scale bar = 50 µm. Significance * *p* < 0.05, ** *p* < 0.01 vs control group and ^#^ *p* < 0.05, ^##^ *p* < 0.01 vs glutamate-injected group. n.s = non-significant difference.

Previous studies have reported that glutamate excitotoxicity increases the expression of phosphorylated JNK (P-JNK) and p38 (P-p38) MAP kinases in culture cells and is associated with cell death and apoptosis [58,59]. In the current study, we found that exogenous glutamate administration in the developing rat brain increased the quantified ratio of P-JNK/total-JNK and P-p38/total-p38, indicating increased phosphorylation of JNK and p38 (Figure 6a–c). The confocal analysis of immunostained brain sections also revealed an upsurge in the number of P-JNK-positive cells stained with DAPI (Figure 6e,f). Moreover, glutamate exposure also caused the downregulation of normalized P-Erk1/2/total-Erk1/2, indicating a decrease in P-Erk1/2 expression (Figure 6a,d). Contrariwise, co-treating E2 with glutamate significantly reduced the quantified ratio of P-JNK/JNK and P-p38/p38 and enhanced P-Erk1/2 when normalized to total Erk1/2 (Figure 6a–d). Furthermore, the E2 + glutamate co-treated brain slices also revealed a low number of P-JNK-positive cells/immunoreactivity in the cortex and hippocampal brain regions (Figure 6e,f). Notably, the E2-alone-treatment did not affect any of the studied parameters when compared to the control group (Figure 6a–f).

Collectively, these results demonstrate that the exogenous glutamate mediates its detrimental effect by triggering the phosphorylation of stress-activated JNK and P38 protein kinases and concomitant inhibition of Erk1/2 phosphorylation to induce apoptosis and cell death, while E2-treatment probably exerts neuroprotection by the induction of the anti-apoptotic pro-survival pathway through Erk1/2 phosphorylation to alleviate neurodegeneration in glutamate-injected postnatal rat brain. 

## 4. Discussion

Glutamate is one of the salient neurotransmitters in the brain. Approximately 90% of neurotransmission occurs through amino acid in which 40% is regulated by glutamate [60]. Glutamate is concentrated in the presynaptic terminal by synaptic vesicles; when the presynaptic membrane depolarizes, it releases glutamate to the synaptic cleft [1]. The excessive production of glutamate leads to loss of function and death of neurons, a process called excitotoxicity [56]. It has been reported that glutamate excitotoxicity is involved in many neurodegenerative diseases, like amyotrophic lateral sclerosis, Parkinson’s disease, and Alzheimer’s disease (AD) [61]. In the current study, we focused on the excitotoxicity of glutamate-induced reactive oxygen species (ROS) production that leads to dysregulation of MAP kinase-mediated neuroinflammation, synaptic dysfunction, and neurodegeneration in the cortical and hippocampal brain region of postnatal day 7 (P7) rats. Moreover, our study also elucidated the neuroprotective role of 17β-estradiol (E2), female sex steroid hormones, against the glutamate-induced detrimental effects in vivo for the first time. Our results make evident that a single glutamate subcutaneous injection (10 mg/kg) induced an intense surge in ROS production and alteration in cellular redox homeostasis. The glutamate-induced oxidative damage was accompanied by the dysregulation in the MAP-kinases pathway, neuropathological inflammation, neuronal degeneration and synapse loss, as revealed through both immunoblot and immunofluorescence analysis. On the contrary, the co-treatment of E2 with glutamate alleviated glutamate-induced discrepancies by reducing brain oxidative stress and by inhibiting P-JNK and P-p38 and by activating Erk1/2 phosphorylation.

Oxidative stress (OS) is a proximal event in many neurodegenerative disease pathogenesis, including AD. The brain cortical and hippocampal regions are most vulnerable to oxidative stress and are associated with the development of synapse/cognitive loss and neurodegeneration [62]. Glutamate toxicity has been implicated in many neurodegenerative diseases, and its excessive production is known to increases ROS production in neurons [63]. It has been reported that the exposure of neuronal cells to exogenous glutamate induces oxidative stress due to the loss of cellular glutathione (GSH) levels and causes mitochondrial dysfunctions [64,65,66,67]. The GSH depletion in neuronal cells alters the intracellular redox state causing accumulations of oxidants [68]. Our present study also provides similar evidence and extent of these findings to the in vivo P7 brain. Our results showed that exogenous-glutamate administration to rat pups altered the brain antioxidant system in both the cortical and hippocampal brain regions by reducing the cellular GSH store and caused an upsurge in ROS and LPO as revealed by their respective assays. Cellular redox dyshomeostasis was accompanied by dysregulation in the Nrf2/HO-1 anti-oxidant signaling pathway. The Nrf2 and its downstream HO-1 inducible antioxidant enzyme have conservative roles against increased oxidative stress, and its dysregulation has been implicated in many neurodegenerative diseases [69,70]. On the contrary, stimulating the Nrf2-ARE pathway in the brain using natural and/synthetic or electrophilic compounds has been considered as one of the major pharmaceutical/therapeutic strategies for preventing and treating neurodegenerative disease [71,72,73,74,75,76]. Our results also revealed that co-treatment of E2, an antioxidant with glutamate, potentially reduced OS by regulating Nrf2-mediated HO-1 and GSH cytoprotective enzyme expression. Previously, we have reported the neuroprotective and antioxidant role of E2 against the increased oxidative stress in the ageing mouse model as well as against ethanol-induced neurodegeneration in the postnatal rat brain [39,77]. Likewise, E2 exhibits intrinsic anti-oxidant activity against numerous stressors in different cell lines [36,37,38]. Moreover, E2 has been reported to increase the antioxidant capability by increasing Nrf2 activity [39,78,79] and by mediating the activities of phase II antioxidant enzymes in the brain [80].

ROS-mediated oxidative stress is known to activate different cellular signaling pathways involving the activation of pro-apoptotic stress-responsive JNK and p38 MAP kinase [15,81]. Both JNK and p38 kinases respond to stress stimuli of different origins, including cytokine stimulation, ionizing radiation and osmotic shock [82]. The glutamate-induced ROS in cultured neuronal cells has been reported to trigger the prolonged activation of MAPKs, leading to cell death [83,84]. Moreover, several reports have demonstrated that the activation of JNK and p-38 suppressed the activity of the Erk MAPK-signaling pathway [85,86,87,88,89,90,91,92,93]. Similarly, Gclm^–/–^ mice, which have depleted GSH levels, have increased phosphorylation of JNK and p-38 paralleled by reduced anti-apoptotic Erk1/2 phosphorylation [94]. Accordingly, in the current study the glutamate-treated P7 pups, which presented enhanced ROS/oxidative damage, were also associated with the increased activation of JNK and p38 phosphorylation and inhibition of Erk1/2 phosphorylation. On the contrary, E2 co-treated with glutamate displayed inhibition of pro-apoptotic JNK and p38 and activation of survival-promoting Erk1/2 MAPK signaling pathways. Erk1/2 is an important signal molecule that regulates multicellular responses to diverse external stimuli, and its activity is crucial for neuronal plasticity, neuronal survival and differentiation [95]. It has been well established that E2 can activate the Erk1/2 MAPK pathway [96,97,98]. Dorsa and colleagues have revealed that E2 can induce the phosphorylation of Erk [37,38,99]. Altogether, these finding demonstrates that E2 confers neuroprotection against glutamate excitotoxicity by promoting the cell survival instinct and opposing the pro-apoptotic activity possibly via regulating the MAP-kinases in the developing rat brain.

Neuroinflammation is a complex phenomenon and is strictly interconnected with excitotoxicity, since glutamate spillover critically favors glial cell activation, promoting brain neuroinflammation [52,100,101]. Both p-JNK and p38 MAP-kinases are critical mediators of inflammation [102,103]. Inhibition of P-JNK activity rescued microgliosis and suppressed neuroinflammation by reducing the expression levels of TNF-α and IL-1β in ischemic stroke [104]. Likewise, p38 MAP-kinase signaling is associated with increased astrogliosis and GFAP protein expression [105] and also regulates chemokine production and the recruitment of activated microglia to the injury site [106]. All of these findings support the current study. Our glutamate-injected P7 pups revealed activated gliosis as indicated by increased GFAP and Iba-1 protein expression. Importantly, E2 reduced the glutamate-induced gliosis and secretion of proinflammatory cytokine (TNF-α). There is a greater consensus on the potential of E2 to confine the glia’s hyper-immune response and exert anti-inflammatory effects [107,108,109]. E2 protects brain neuronal cells from prolonged inflammation by attenuating microgliosis [40]. Moreover, treatment with E2 can suppress LPS-induced inflammation and cytokine production in microglia [110,111,112]. Similarly, ovariectomized (OVX) mice, when treated with E2, suppressed gliosis within the hippocampal-DG and CA region [113]. Together, these results illustrated that E2 can protect the brain from glutamate toxicity by mitigating gliosis and the release of pro-inflammatory cytokines.

**Figure 7:**
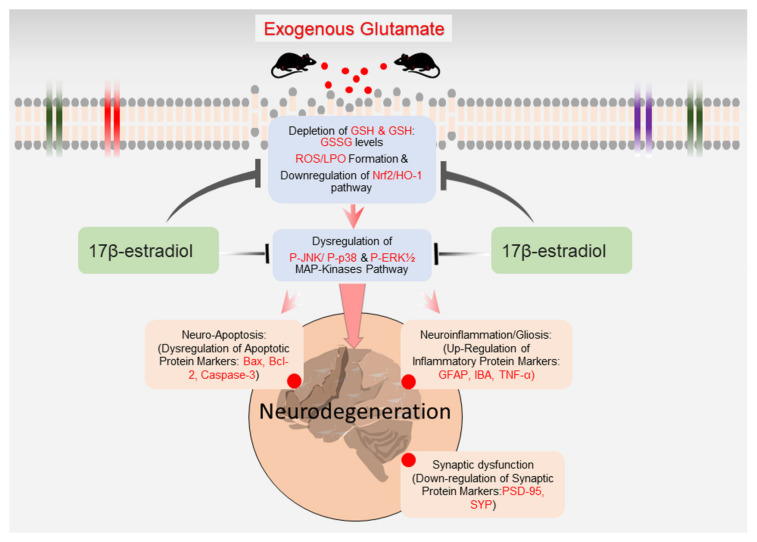
The graphical representation of the neuroprotective mechanism of estradiol against glutamate-induced neurodegeneration in developing rat brain. Exogenous glutamate (single subcutaneous injection of 10 mg/kg) treatment increased brain oxidative stress associated with the dysregulated MAP kinase pathway mediated neuroinflammation, synapse loss and neurodegeneration in postnatal 7-day rat brain. Co-administration of 17β-estradiol with glutamate alleviated glutamate-induced neurodegeneration in the developing rats.

Excitotoxicity is a complex phenomenon contributing to synaptic loss and neurodegeneration [1,114,115]. The role of p38 MAPK pathway-induced cytokine production leading to synapse loss and neurodegeneration has been extensively reviewed [116]. Inhibiting p38 MAPK activity suppresses brain neuroinflammation and attenuates synaptic dysfunction and neurodegeneration in the AD model [117]. The activation of the p-JNK/p38 pathway, also called a death pathway, negatively regulates the P-Erk pathway to induce the apoptotic signal [85,90,94,118]. The dysregulation in MAP-kinases upregulates Bax and caspase-3 and downregulates the anti-apoptotic Bcl-2 protein expression [119,120]. Our data also revealed the downregulation of the synaptic (PSD95 and SYP) and dysregulation of the expression of the pro-apoptotic (Bax, Bcl2 and caspase-3) protein associated with the abnormal intermediation of MAP-kinase pathway in glutamate-treated pups. Importantly, E2 co-administered with glutamate improved synapse density and rescued the cortical and hippocampal neurodegeneration. CNS disorders associated with neurodegeneration have a huge impact on healthcare systems [121]. Accumulative evidence suggests that sex is a significant variable in the prevalence and incidence of neurodegenerative disorders like AD, MS and Parkinson’s disease and, personalized treatment [122,123,124,125]. Recent studies have revealed that in male multiple sclerosis (MS) patients, estrogen synthesis and signaling are induced, while in female MS patients, progestogen synthesis and signaling are induced that may affect lesion pathogenesis [123]. Moreover, it has been reported that old postmenopausal and perimenopausal women exhibit neurodegenerative phenotypes associated with a decrease in the ovarian secretion of estrogen and progesterone [126,127]. This decline in estrogen levels contributes to dysregulated glucose metabolism in different brain regions that confer cognitive functioning and synaptogenesis [128]. Likewise, the OVX Sprague—Dawley rats reveal a neurodegenerative phenotype and are commonly used to mimic the pathological changes of post-menopausal females [129,130,131]. Importantly, replacing estradiol levels with hormonal therapy in OVX rats improved mitochondrial function and rescued neurodegeneration [131,132,133]. Similarly, E2 has been reported to increase synaptic density [134,135] and is widely known to induce synaptogenesis [135,136,137,138]. Altogether, these data demonstrate that E2-treatment could alleviate glutamate-induced synapse and neuronal apoptosis. We suggest a simple schema to illuminate how E2 may protect against glutamate-induced oxidative damage, neuroinflammation and neurodegeneration (Figure 7).

## 5. Conclusions

Collectively, these data demonstrate that glutamate-induced oxidative stress (ROS) mediates neuronal loss/induction of apoptosis by the persistent activation of the JNK/p38 pathway and by suppressing the survival-promoting Erk1/2 MAP-kinase pathway. Similarly, we elucidated for the first time in vivo that 17β-estradiol can reverse the glutamate-induced detrimental effects, likely by activating the expression of the cytoprotective enzyme by activating the Nrf2/HO-1 antioxidant and regulating MAPKs pathways. The study also demonstrates that 17β-estradiol is a highly potent agent against glutamate-induced neuroinflammation, synapse loss and neurodegeneration. Future mechanistic studies are warranted to illustrate the detailed molecular mechanism of 17β-estradiol neuroprotection.

## Figures and Tables

**Table 1:** List of primary antibodies and their information used in this study.

Antibody	Catalog/Product #	Application (Conc.)	Host	Manufacturer
β-Actin	SC-47,778	WB (1:1000)	Mouse	Santa Cruz Biotech
Nrf2	SC-722	WB/IF (1:1000)	=	=
HO1	SC-136,961	WB/IF (1:1000/1:100)	=	=
GFAP	SC-33,673	WB/IF (1:1000/1:100)	=	=
Iba-1	SC-32,725	WB (1:1000)	=	=
Iba-1	PA5-27,436	IF (1:100)	Rabbit	Thermo Fisher
TNF-α	SC-52,746	WB (1:1000)	Mouse	Santa Cruz Biotech
PSD-95	SC-71,933	WB (1:1000)	=	=
SYP	SC-17,750	WB (1:1000)	=	=
Bax	2772S	WB (1:1000)	Rabbit	Cell Signaling
Bcl-2	SC-7382	WB (1:1000)	Mouse	Santa Cruz Biotech
Caspase-3	SC-7272	WB (1:1000)	=	=
Caspase-3	9661S	IF (1:100)	Rabbit	Cell Signaling
P-JNK	SC-6254	WB/IF (1:1000/1:100)	Mouse	Santa Cruz Biotech
JNK	SC-7345	WB (1:1000)	=	=
P-p38	#9212	WB (1:10,000)	Rabbit	Cell Signaling
p38	#9211s	WB (1:1000)	=	=
P-Erk1/2	#9101	WB (1:10,000)	=	=
Erk1/2	9102S	WB (1:10,000)	=	=

## Data Availability

The authors hereby declares that the data presented in this study will be presented upon request from the corresponding author.

## Abbreviations

OS	Oxidative stress
ROS	Reactive oxygen species
E2	Estradiol
Glu	Glutamate
LPO	Lipid peroxidation
Nrf2	Nuclear factor erythroid 2-related factor 2
HO-1	Heme oxygenase-1
p-JNK	c-Jun n-terminal kinase
P-p38	phosphorylated p38
P-Erk1/2	Extracellular signal-regulated kinase 1 and 2
GFAP	Glial fibrillary acidic protein
Iba-1	Ionized calcium-binding adaptor molecule 1
TNF-α	Tumor necrosis factor alpha
Bax	Bcl-2-associated X protein
Caspas3	cysteine-aspartic acid protease 3
Bcl-2	B-cell lymphoma 2
